# Attention Span of Children With Mild Intellectual Disability: Does Music Therapy and Pictorial Illustration Play Any Significant Role?

**DOI:** 10.3389/fpsyg.2021.677703

**Published:** 2021-05-11

**Authors:** Udeme Samuel Jacob, Jace Pillay, Esther Olufunke Oyefeso

**Affiliations:** ^1^Postdoctoral Research Fellow, South African Research Chair: Education and Care in Childhood, Faculty of Education, University of Johannesburg, Johannesburg, South Africa; ^2^Department of Special Education, Faculty of Education, University of Ibadan, Ibadan, Nigeria; ^3^South African Research Chair in Education and Care in Childhood, Faculty of Education, University of Johannesburg, Johannesburg, South Africa

**Keywords:** attention span, children, mild intellectual disability, music therapy, pictorial illustration

## Abstract

This study investigated the effects of music therapy and pictorial illustration on the attention span of children with mild intellectual difficulties. A pre-test, post-test and control group quasi-experimental research design was used with a sample of children diagnosed with mild intellectual disability from three special schools in Ibadan, Nigeria. Fifty children were randomly selected and assigned to one of three groups: music therapy, pictorial illustration, or control (*N* = 50, male = 25, female = 25, mean age = 11.6 years). Twenty-four sessions of music therapy and pictorial illustration classes were held with the experimental group only. The Moss Attention Rating Scale was used before and after the intervention to collect data on participants’ attention span. Analysis of Covariance indicated that there was a significant statistical difference between pre-test and post-test results of the two groups. The estimated marginal means of post-attention span by treatment indicated that pictorial illustration had the highest post-attention span score, followed by music therapy, while post-attention span score for the control group was the least. Based on the findings, it is recommended that teachers, caregivers, and parents of children with mild intellectual disability adopt pictorial illustration and music therapy as teaching strategies to enhance their attention span.

## Introduction

A significant challenge faced by children with intellectual disability is the difficulty of paying attention to classroom instruction long enough to acquire the information they need to effectively participate in learning activities. Most children with intellectual disability have the ability to complete academic and non-academic tasks, but their inability to pay attention to detailed instruction frequently leads to failure. To ensure that the skills required to complete assigned responsibilities are easily developed, it is important that appropriate teaching strategies are used to enhance the attention of children with intellectual disability during teaching and learning process (Deutsch et al., 2008). Attention span among school-aged children is particularly crucial, as active participation (on-task and on-schedule behavior) in academic activities can contribute to improved academic performance (Carnahan et al., 2009). The term “attention” refers to the ability of an individual to concentrate their perception and thought on a specific task while deliberately ignoring extraneous stimuli (Erbay, 2013).

Attention-related issues have been, or may be, due to learning disabilities, or triggered by them. More information on attention span among children with developmental disabilities is required due to the co-morbidity of intellectual disability and attention deficit hyperactivity disorder (ADHD) (American Psychiatric Association, 2013). Various studies have shown that the child’s ability to pay attention explicitly predicts academic performance. The level of children’s attention functionality, according to Commodari (2012), correlates with the likelihood of developing learning disorders in a learning environment. Moreover, attention span has significant correlation with the overall achievement of a child later in life (Commodari, 2012; Gardner-Neblett et al., 2014). Attention deficit is often associated with impaired mental skills and weak impulse and behavioral control, leading the child to participate in off-task and/or destructive behavior at school (Commodari, 2012; Gardner-Neblett et al., 2014).

An alerting cue can be used to establish the appropriate level of attention (Hedges et al., 2013). A child may process information efficiently using the alerting cue while their attention is focused on appropriate tasks. In essence, the relationship between attention of children with intellectual disability and the use of alerting cues should be investigated to determine their efficacy. While interventions to enhance attention span in children have been identified, most of these are ineffective for children with intellectual disability because they lack the cognitive skills to function in certain circumstances. Among the intervention strategies are visual materials, promoting predictability using routines, the use of self-discipline, music, personality-reliance instruction, applied behavior analysis, and prescription drugs (Deutsch et al., 2008; Carnahan et al., 2009; Gardner-Neblett et al., 2014). The ineffectiveness of such interventions may also be attributed to the inability of children with intellectual disability to recall information previously learnt.

For most children with intellectual disability, delays in visual processing, language processing, and comprehension of basic English are likely to lead to difficulties sustaining the required level of attention for learning. The objective of this research was to investigate the effects of music therapy and pictorial illustration on the attention span of children with mild intellectual disabilities.

## Literature Review

### Benefit of Attention on Classroom Participation

Children with intellectual disability often fail to follow instructions and seem not to be listening when directly spoken to Christiansen et al. (2015). They may demonstrate a reluctance to participate in activities that involve sustained mental energy or close attention to detail and may make careless mistakes in their work. The primary importance of attention span is to enable the child with intellectual disabilities to focus on the task for a designated amount of time (Diamond and Lee, 2011). Teachers suggest that listening to each word during the learning process makes it easier for children to develop a deeper and better understanding of classroom instruction and concepts, and reduces the time needed for revisions of what was learned afterward. Generally, teachers give their pupils this information without understanding how factual it is, or even if it is true.

Attention plays a crucial role in different cognitive activities (Gray et al., 2003), for example, spatial cognition, comprehension, long-term memory, logical thinking, and general executive functioning (Dean, 2006). Typically, a person becomes aware of the characteristics of an object when attention is paid to those objects, and the objects will fade away from consciousness as soon as attention is transferred to other objects (Laureys and Tononi, 2009). Nevertheless, Shelton et al. (2008) do not agree with the submission based on their perception that not all activities being focused on can be processed by the conscious mind, or vice versa. Schweizer (2010) argues that there are various types of attention, including: sustained attention, which involves a great deal of continuous mental effort; selective attention, also defined as intense attention; and managed attention, which refers to the controlled processing of information.

Attention is crucial part of learning. It is believed that paying attention to classroom activities can enhance the learning process, as attending lessons has a significant impact on the immediate response of pupils (Kruschke, 2005). Selective attention has been described as the most valuable aspect of learning, since it allows pupils to concentrate only on what is relevant in the learning environment (Kruschke, 2005). Children with intellectual disabilities are likely to face several additional obstacles that may distract them from learning effectively, such as the classroom arrangement, the school culture, noises from both within and outside the classroom, and the teacher’s voice and teaching style. The reason why children with intellectual disability should be trained to avoid all forms of classroom distractions is that it can impede the efficient processing of information in such a learning environment.

### Music Therapy

Music therapy, consisting of listening to or performing music often as a group, has been ranked among the most highly ranked forms of complementary and alternative treatments (Klein and Kemper, 2016). Therefore, music is particularly important as an avenue of social contact and non-verbal self – expression (Johnels et al., 2016). Findings have shown that the use of music as a teaching strategy is efficient in enhancing academic achievement in a variety of subject areas (Iwasaki et al., 2013). This is due to the rhythm and appealing qualities of the music, choosing songs that naturally attract the children’s attention. Eren et al. (2013) and Constanti (2015), for instance, discussed the effect of music among children with special needs. Their findings inferred that music potentially reduced anxiety levels and stereotypical behaviors of preschoolers with special needs. Other research outcomes are convergent toward enhancing communication skills, attention span, and increasing regulation of emotions (Gooding, 2011; Nguyen, 2014).

In the submission of Kern et al. (2007), children with autism can be taught to follow classroom routine using music therapy. Sze (2006) opined that music can be incorporated into literature to assist with content engagement and to help children interact with, and participate in, specific tasks. Different researchers have focused on using music intervention to improve learning among children, irrespective of their level of ability. Songs with repetitive lyrics can be efficiently used as teaching techniques for the development of reading fluency (Iwasaki et al., 2013). Sze (2006) noted that music helped with cognitive development because of its predictable organization. In addition, according to Sze, “the integration of music provides children with tangible, hands-on interactions that are vital to the development of each child’s ability to comprehend, evaluate, solve problems, assess, and promote creativity” (2006, p. 2). Psychosocial, emotional, cognitive, and communicative disorders have been successfully treated with music therapy (Baker et al., 2008). A well-known type of music therapy is Nordoff-Robbins.

In this type of music therapy, improvisation is used by therapists to make up songs that assist the client work through what they feel at a particular moment (Sorel, 2010). The use of music therapy for children with intellectual disability can help facilitate spontaneous non-verbal communication and can also teach basic skills, like reading (Register et al., 2007; Markworth, 2014). Most songs used for music therapy begin with “hello” and end with “good-bye,” which establishes a routine and expresses welcome. This demonstrates that an ideal starting point to teach children with disability how to keep routines is by using music therapy (Kern et al., 2007). Carnahan et al. (2009) argued that music is efficient in enhancing participation, processing, and retention of information, and that it can also improve students learning behavior. An investigation of the impact of visual aids paired with music on the participation of students with autism during small group instruction revealed that the use of visual aids paired with music resulted in an increase of students’ participation (Carnahan et al., 2009).

### Pictorial Illustration

The combination of texts with images is generally perceived to have a positive effect on children’s learning and attention. For example, pictorial illustrations are often used as a way of inspiring readers by making texts more appealing and interesting (Male, 2007). Not only is retention of information enhanced by pictorial illustration, but it also strengthens the ability to comprehend complex subjects that ultimately contribute to the development of appropriate problem-solving skills, provided text and images are compatible and spatially or temporally similar (Mayer, 1997, 2009; Moreno and Mayer, 1999). From a cognitive point of view, pictorial illustrations are intended to enhance ability based on the use of the multimedia effect as they present relevant information in a visual form and provide clear support for mental models of construction (Schnotz and Bannert, 2003; Schnotz, 2005; Mayer, 2009). It can be concluded that the more attention the images attract, the less distracted from learning a learner would be (Harp and Mayer, 1998; Sanchez and Wiley, 2006).

The use of pictorial illustration as a resource is common in primary school because it can help to resolve and increase understanding of concept taught and acceptance of disability among learners. Beyond learning skills through text and pictures in children’s books, pictorial illustration can serve as a foundation for developing children’s self-awareness and helping them to understand the culture of others around them (Cole and Valentine, 2000). The use of picture books can play a key role in the education of children to enhance learning outcomes (Golos et al., 2012). Koss (2015) also consider pictorial illustration to be one of the most important mediators for transmitting cultural awareness and social values to children, as well as an interesting tool to understand the world and solve complex problems that could define the reality in which each child lives. The characters in the illustrations can also help to enhance learning atmosphere by emphasizing diversity, social equality, tolerance, and empathy for learners with disabilities (Sigmon et al., 2016). It can, however, be a challenging process to select and use appropriate books to effectively facilitate understanding among children with special needs, particularly those with intellectual disability (Ostrosky et al., 2013).

## Methodology

### Hypothesis

The null hypotheses tested at 0.05 level of significance was: There is no significant main effect of treatment (music therapy and pictorial illustration) on enhancing attention span of participants.

### Participants

Fifty children with mild intellectual disability were selected for the study. They were selected using a multi-stage sampling method from three special schools to reflect geographical coverage of the Ibadan metropolis, Oyo State. A purposive sampling technique was used for the selection of pupils with moderate intellectual disability based on assessment by a psychologist. Participants were assigned randomly to treatment groups tagged as M, P, and C, representing the treatment they were exposed to. In school M, out of a population of 32 pupils, 15 with mild intellectual disability (*N* = 15, male = 8; female = 7; mean age = 11.8) were selected; in school P, out of a population of 40 pupils, 18 with mild intellectual disability (*N* = 18, male = 8; female = 10; mean age = 11.3) were selected; and in school C, out of 38 pupils, 17 with mild intellectual disability (*N* = 17, male = 9; female = 8; mean age = 11.6) were selected. Participants in school M were exposed to music therapy, those in school P were exposed to pictorial illustration, and participants in school C were exposed to conventional methods of teaching, thereby serving as the control group. The reason for not exposing children from the same school to the three treatments was to avoid contamination.

### Materials and Methods

The study was based on a quasi-experimental research design of the pre-test control group using a factorial matrix of 3. Three levels of treatment (music therapy, pictorial illustration, and control) were considered. The design is represented thus:

Experimental Group 1: (E1) O_1_ X_1_ O_4_

Experimental Group 1: (E1) O_2_ X_1_ O_5_

Control Group 2: (E2) O_3_ (E2) O_6_ O_3_ O_6_

Where:

O_1_, O_2_, and O_3_ represent experimental and control group pre-test scores, respectively.

O_4_, O_5_, and O_6_ represent experimental and control group post-test scores, respectively.

X_1_ represents the treatment for experimental group (the teaching mode of music therapy).

X_1_ represents the treatment for experimental group (the teaching mode of pictorial illustration).

### Music Therapy

Traditional folk songs were used for the music therapy. Each session was held using drum sets, a piano and recorders. The teacher sang the song at the start of each therapy session with the support of a music therapist, and the lyrics explained to participants what attentive behaviors were expected (appropriate vocalizations, eyes on speaker/materials, appropriate hand movement, sitting upright, and still feet). The music therapy treatment consisted of twenty-four sessions of 45 min each for the treatment group. Participants were encouraged to sing along and dance to the music. Participant names were inserted into the songs and when necessary specific actions was taken by the participants such as dancing, stamping of the feet among others. For the control group, no intervention was used, and they were not asked to attend any music therapy sessions. Example of the sing used includes:

Bàtà mi á dún ko ko kà – My shoes will make a rich sound.

Lábẹ́ì igi òroẹ́bó |- Under the orange tree.

### Pictorial Illustration

During the pictorial illustration sessions, images and diagrams were drawn by hand. Each session was held using drawing books, pencils, and crayons. The pictorial illustration treatment consisted of twenty-four of 45 min each for the treatment group. Participants were encouraged to draw pictures representing their ideas and responses to questions. For the control group, no intervention was used, and they were not asked to attend any pictorial illustration sessions.

### Moss Attention Rating Scale

The Moss Attention Rating Scale was designed in 2003 by John Whyte and colleagues. The scale has 22 items assessed on a five-point Likert-type scale based on how well an individual behavior is represented. The response ranged from “definitely true” to “definitely false.” The phrases used for each item were such that participants were expected to perceive each behavior as an indication of ether acceptable or inappropriate attention. Most items had a relationship with the three predictor variables observed for the Moss Attention Rating Scale (MARS): restlessness, distractibility, initiation, and sustained consistent attention (Hart et al., 2006). The total raw MARS score may be converted to an interval metric from 0 to 100. The MARS has been subjected to two large sample studies of inter-rater reliability. The first (Whyte et al., 2003) compared ratings of patients’ occupational therapists (OTs) and physical therapists (PTs) done independently over the same 3-day window. The MARS was originally designed as an observational rating scale to provide a reliable (*r* = 0.64), quantitative, and ecologically valid measurement of attention-related behavior after traumatic brain injury (TBI) (Hart et al., 2009). The scale was pilot tested to determine it suitability for pupils with mid intellectual disability. It yielded a cronbach’s alpha of 0.69.

### Ethical Consideration

Participant’s parents were duly informed about the aim of the study by through a letter sent to them and required to be at the school on a specific day with the researcher. In line with the ethics of research, the researcher a meeting was held with participant’s parents where the content of the consent form was explained to them by the a teacher who also serve as research assistant, using native language. Once adequate understanding was ensured, each parent completed and appended their signature to the consent form. Participants were assured of the confidentiality of their profiles and responses. One major restriction in the study was that the sessions was not recorded.

### Procedure

The local education authority granted approval to conduct the study and were informed of its purpose. An introduction was presented to the head of the selected school. Three research assistants served as resource teachers for the three groups. Teacher of children with special needs implemented the music therapy while trained therapist assisted so that it can be appropriately carried out. They were trained during sessions of 5 h each for 3 days on how to provide the intervention to participants. After that, the attention span scale was administered to selected children with intellectual disability. The data collected was the pretest score. The intervention lasted for 8 weeks, with 45 min sessions. Placebo treatment was given to the control group (conventional method). The instrument used during the pretest was also used to evaluate the treatment and control group as the post-test after the 8 weeks of intervention.

### Data Analysis

The data generated were analyzed using Analysis of Covariance (ANCOVA) to test the null hypotheses at 0.05 level of significance difference. Moreover, the estimated marginal means and Bonferroni *post hoc* results were determined.

## Results

H_*o*__1_: There is no significant main effect of treatment on attention span of children with intellectual disability.

Table 1 shows that there was significant main effect of treatment in enhancing attention span of children with intellectual disability [*F*_(__2_,_35__)_ = 443.582; *p* < 0.05; partial η^2^ = 0.962]. The effect size is 96.2%. This implies that 96.2% variance in the post-reading performance of learners with intellectual disability was accounted for by treatment, hence, there was a significant difference in the attention span of children with intellectual disability Therefore, the null hypothesis was not accepted. The level of significant main effect was determined groups using estimated marginal means of the treatment groups and the result is presented in Table 2.

**TABLE 1:** Analysis of covariance of post-attention span of children with intellectual disability by treatment.

Source	Type III sum of squares	Df	Mean square	*F*	Sig.	Partial eta squared
Corrected model	1979.691	8	247.461	139.207	0.000	0.970
Intercept	143.576	1	143.576	80.767	0.000	0.698
Pre-test	68.723	1	68.723	38.659	0.000*	0.525
Treatment	1577.064	2	788.532	443.582	0.000*	0.962
Error	62.218	35	1.778			
Total	31890.000	44				
Corrected total	2041.909	43				

*Significant.

**TABLE 2:** Estimated marginal means of post-attention span by treatment.

Treatment	Mean	Std. error	95% confidence interval	
			Lower bound	Upper bound
Music therapy	24.160	0.310	23.530	24.789
Pictorial illustration	34.076	0.406	33.253	34.900
Conventional	14.373	0.478	13.402	15.343

Table 2 shows that the learners with intellectual disability in the pictorial illustration group had the highest post-attention span score (34.076), followed by music therapy (24.160), while the students in the conventional group had the lowest mean score (14.373). This order could be represented as PI > MT > CS.

Table 3 shows that there was a significant difference in the post-reading performance of students in the music therapy and pictorial illustration groups (Mean difference = −9.916; *p* < 0.05); music therapy and conventional (Mean difference = 9.787; *p* < 0.05); and pictorial illustration and conventional (Mean difference = 19.704; *p* < 0.05).

**TABLE 3:** Bonferroni *post hoc* analysis of the reading performance by treatment.

(I) Treatment	(J) Treatment	Mean difference (I-J)	Std. error		Sig.^*d*^	95% confidence interval for difference^*d*^
					Lower bound	Upper bound
Music therapy	Pictorial illustration	−9.916*	0.521	0.000	−11.226	−8.607
	Conventional	9.787^∗^	0.563	0.000	8.370	11.204
Pictorial illustration	Music therapy	9.916*	0.521	0.000	8.607	11.226
	Conventional	19.704^∗^	0.642	0.000	18.090	21.317
Conventional	Music therapy	−9.787^∗^	0.563	0.000	−11.204	−8.370
	Pictorial illustration	−19.704^∗^	0.642	0.000	−21.317	−18.090

* indicate result is significant.

### Discussion of Findings

Based on the hypothesis, the results show that treatment (music therapy and pictorial illustration) had a significant effect on the attention span of children with intellectual disabilities. This is consistent with the suggestion of Sze and Yu (2004) that integrating music and learning for students could provide opportunities for realistic, hands-on interactions that are important for the development of each student’s ability to reason, reflect and resolve conflicts. This shows that that music can be used effectively for the management of medical and psychological challenges. Furthermore, music therapy as an intervention, it has improved motor performance in children and coordination and strength in children with ID (Ekins et al., 2019). The intervention group of the present study participated in a multidimensional movement of activity during the intervention that stimulated all sensory systems.

These findings are consistent with recent studies suggested that regular physical activity programs such as music that will allow participants to move their body had positive effects on the social and school behavior characteristics of individuals with Intellectual Disabilities (Best, 2010; Brooks et al., 2015; Güvendi and İlhan, 2017; Yílmaz and Soyer, 2018). Moreover, present finding on the effect of pictorial illustration on attention span aligns with previous research submission that a temporal relationship exist between information processed visually and verbally (Hill, 2003; Butcher and Vincent, 2007; Özdemir, 2007). In the opinion of Bruscia (2014) music centered interactions are an undeniable aspect of music therapy, where music is the acting motivator because music contributes to therapy as a tool, a process and an outcome.

Furthermore, the estimated marginal means of post-attention span by treatment revealed that pictorial illustration had the highest post-attention span score, followed by music therapy, while the control group had the lowest post-attention span score. The present findings are consistent with the report of Ryu, cited in Roslina (2017), that using dual coding theory (visual and verbal mode) for processing information constitute a challenge, when the system to process the information is more than one. This shows that it is more efficient using two sources of information during classroom activities because it will enhance attention span required for improving comprehension. Moreover, pictorial illustration was used as a means of motivating and engaging pupils, thereby increasing student achievement. The estimated marginal means for music therapy is not consistent with the submission of Anderson and Fuller (2010), who reported a significant decline in reading comprehension after pupils listened to lyrical music, compared to the control group that read in a quiet environment. The result is, however, consistent with the previous report that music has a positive effect on increasing on-task behavior (Dieringer et al., 2017) and decreasing off-task behavior (Titus and Porretta, 2012) for children with ASD.

## Conclusion and Recommendations

Based on the findings of the study, it can be concluded that treatment (music therapy and pictorial illustration) had a significant effect on the attention span of children with intellectual disability when compared to participants in the control group. The reason for the effectiveness of music therapy could be due to the active participation of learners in singing the songs (traditional folk songs were used) and in that way the interest of the learners was better sustained in comparison to participants in the control group. Pictorial illustration was the more efficient of the two treatments in sustaining the attention of children with intellectual disability. This was probably due to the opportunity created for the learners to interact with one another while learning through pictures. Music therapy and pictorial illustration are learner-centered instructional strategies which will increase the active participation of learners in any learning environment.

The implication of these findings is that the use of the two strategies (music therapy and pictorial illustration) to enhance the attention of children with intellectual disability should be encouraged among their teachers. This is because both strategies enhance the attention of children with intellectual disability required for active classroom participation. In that way, learning is made concrete, auditory, and visual. Since pictorial illustration had a more significant effect in enhancing the attention span of children with intellectual disability, teachers should consider their use to encourage learners’ active participation in learning tasks. It is, therefore, recommended that stakeholders such as teachers, caregivers, and parents of children with intellectual disability, should be trained on how pictorial illustration and music therapy can be applied to attract the attention of children with intellectual disability.

## Limitations

The small sample size was one of the study’s limitation. Future research should include a larger number of participants. Due to the unpredictability of a variety of factors, conducting the study in a school setting was also a limitation. These factors include participant absences, pupil behavior (which can sometimes be distracting to other pupils and necessitate the removal of staff from the academic setting to deal with behaviors), and scheduled days off from school. Another limitation to the study is one person serving as assessor of the participants’ attention-related behavior in each treatment group. This did not allow for opportunity to compare the degree of agreement or disagreement of observed behavior.

## Data Availability Statement

The original contributions presented in the study are included in the article/supplementary material, further inquiries can be directed to the corresponding author/s.

## Ethics Statement

The studies involving human participants were reviewed and approved by the University of Ibadan. Written informed consent to participate in this study was provided by the participants’ legal guardian/next of kin.

## Author Contributions

UJ conceived the research and did the data collection. JP and EO worked and edited the manuscript. All authors contributed to the article and approved the submitted version.

## Conflict of Interest

The authors declare that the research was conducted in the absence of any commercial or financial relationships that could be construed as a potential conflict of interest.
